# The Fight against Tuberculosis

**Published:** 1920-12-11

**Authors:** 


### THE PUBLIC WELL-BEING.

### The Fight against Tuberculosis.

### WHAT LONDON IS DOING.

A writer in the *International Journal of Public Health* finds encouragement in the gradually decreasing rates of tuberculosis mortality. It is true that since the war the mortality curves have gone up again, but it is claimed that this is due to economic factors, and that the technical medical organisations do their best with the limited public and private resources at their disposal. Dr. F. N. K. Menzies, in his report to the London County Council, hardly seems to share this view, and he has permitted the Journal to make use of the material in his report so as to give greater publicity to the experience he has acquired.

The population of the County of London was estimated at 4,400,164 by the last official census of 1911, and at 4,540,062 in 1918. Among this population the number of deaths from pulmonary tuberculosis, and the number of primary notifications of cases of tuberculosis reached the following figures:—

| | Pulmonary tuberculosis | Other tuberculosis | Total | Primary notifications |
|---|---|---|---|---|
| 1916 ... | 6.491 | 1.514 | 8.505 | 17.631 |
| 1917 ... | 6.908 | 1.575 | 8.483 | 18.735 |
| 1918 ... | 7.048 | 1.398 | 8.445 | 19.178 |
| 1919 ... | 5.332 | 992 | 6.324 | 15.587 |

From these figures it is calculated that the approximate number of tuberculous persons in London, taking for granted that the disease lasts five years, is from 70,000 to 80,000. The estimate is not altogether reliable, owing to the insufficient accuracy of primary notifications and of the duration of the disease. For instance, in schools alone, 827 unreported tuberculous children were discovered only by school inspection.

Vienna!

The figure given is sufficiently appalling, yet it indicates a remarkably low morbidity. It already represents a certain decrease as compared with the war period, and especially with other cities, such as Vienna. The last reports for this city seem to show for 1918 an increase of 113 per cent. over the normal number of deaths from tuberculosis. Among the multiple causes of the increase of tuberculosis in Belgium, attention is called to the very acute state of exhaustion from which deported civilians suffered in Germany. Forty-two per cent. of them had become tuberculous when they returned home.

Deviation from the preventive rôle and the enormous development of the therapeutic aspect of the dispensary constitute, it is alleged, a serious tactical mistake. The dispensary, it is urged, must ferret out the tuberculous entity at all its stages and through all its manifestations. Thanks to the dispensary, not a single case of tuberculosis should occur unobserved or remain uncared for by the community. Once the tuberculosis officer gets in touch with an individual patient he never loses hold of him. He observes the patient himself, and has him sent to the hospital or institution best fitted for his case. He organises the after-care of patients who have left the hospitals, and, above all, he seeks to discover tuberculosis in its recognised haunts. His function requires him to seek out the patient in his home, to get at the "tuberculous focus," to investigate faulty environment, and to search out tuberculosis among other members of the household. He looks after every case that comes to his knowledge, both in the interest of the patient and for the common welfare.

This is declared to be the weak point in the present organisation, the very small number of serious cases of phthisis known to the dispensaries being a striking fact. Dr. Menzies notes that tuberculosis officers complain of not being in touch with health officials. Private dispensaries especially are not kept informed as to notifications of new cases and deaths from tuberculosis occurring in the district. An agreement should also be entered into between dispensaries for the transfer of the records of patients changing their residence. While close relationship with school medical officers would be very fruitful, such relationship, as a matter of fact, "does not usually exist." It is therefore suggested that the tuberculosis officer be made the necessary assistant of the borough medical officer of health. The relationship between the two would then become friendly and closer professionally, and the public resources would be co-ordinated. Dispensaries would be able to study social conditions in their district, and the tuberculosis officer would acquire the administrative experience which he often lacks. Above all, he would be better able to make a thorough search of the tuberculous environment, which is a considerable task.

The Cost of Efficiency.

With regard to institutional treatment, the waiting period is too long, as is the case nearly everywhere, this period being from seven to fifteen days for an ex-soldier, twenty-eight days on the average for a civilian adult, and *eight to twelve weeks* for a child. A critical examination of the London boroughs has shown that they have from 37 to 455 deaths from tuberculosis, and that, on the other hand, a single physician can do useful work only if he has charge of a population with not more than 120 such deaths in a year. For the sake of economy and convenience a maximum figure of 160 might be accepted. On that basis London would require, not thirty-two, but fifty whole-time tuberculosis

**THE PUBLIC WELL-BEING**—(*continued*).

officers—that is to say, an average of one per 90,000 inhabitants. Their work would be enormous even then, as they would each have to supervise 500 to 600 consumptives and their contacts, or possibly 1,000 patients. Compared to the enormous increase of efficiency that would be obtained by better organisation, the increase in cost is relatively insignificant. With a total expenditure of £391,000, of which the City itself would only pay, in round numbers, £120,000 (that is, 1s. to 6s. 1d. per head), thanks to support from the State, hospitals, and the Insurance Committee, it would be possible, it is argued, to get far better and more lasting results through a more intelligent application of purely preventive methods. This estimate apparently does not take into consideration the provision of open-air schools, though we are glad to note that that important factor is not ignored in the preventive programme.

